# Loss of Blood-Brain Barrier Integrity in a KCl-Induced Model of Episodic Headache Enhances CNS Drug Delivery

**DOI:** 10.1523/ENEURO.0116-18.2018

**Published:** 2018-07-16

**Authors:** Karissa E. Cottier, Emily A. Galloway, Elisa C. Calabrese, Margaret E. Tome, Erika Liktor-Busa, John Kim, Thomas P. Davis, Todd W. Vanderah, Tally M. Largent-Milnes

**Affiliations:** 1 Department of Pharmacology, College of Medicine, University of Arizona, Tucson, AZ 85724

**Keywords:** blood-brain barrier, CNS, cortical spreading depression, drug delivery, headache, triptan

## Abstract

Cortical spreading depression (CSD) in the CNS is suggested as a common mechanism contributing to headache. Despite strong evidence for CNS involvement in headache disorders, drug development for headache disorders remains focused on peripheral targets. Difficulty in delivering drugs across the blood-brain barrier (BBB) may partially account for this disparity. It is known, however, that BBB permeability is increased during several CNS pathologies. In this study, we investigated BBB changes in response to KCl-induced CSD events and subsequent allodynia in rats. Cortical KCl injection in awake, freely moving rats produced facial allodynia with peak intensity between 1.5 and 3 h and CSD induction within 0.5–2 h postinjection. Brain perfusion of ^14^C-sucrose as a marker of BBB paracellular permeability revealed increased leak in the cortex, but not brainstem, beginning 0.5 h post-KCl injection and resolving within 6 h; no changes in tight junction (TJ) proteins occludin or claudin-5 expression were observed. Acute pretreatment with topiramate to inhibit CSD did not prevent the increased BBB paracellular permeability. CNS delivery of the abortive anti-migraine agent sumatriptan was increased in the cortex 1.5 h post-KCl injection. Surprisingly, sumatriptan uptake was also increased in the brainstem following CSD induction, suggesting regulation of active transport mechanisms at the BBB. Together, these results demonstrate the ability of CSD events to produce transient, time-dependent changes in BBB permeability when allodynia is present and to mediate access of clinically relevant therapeutics (i.e., sumatriptan) to the CNS.

## Significance Statement

The need to overcome the blood-brain barrier (BBB) is one of the major hurdles in designing CNS active drugs. CNS mechanisms have been shown to play a vital role in primary and secondary headache disorders. In this study, we show that BBB permeability is transiently increased in a rat model of cortical spreading depression (CSD)-induced episodic headache. We also observed a significant increase in sumatriptan uptake into the brain. Clinically, sumatriptan has been shown to be most efficacious when given close to the onset of headache pain, corresponding to times where we observed increased CNS uptake in our rat model. These data suggest that altered BBB permeability occurs during CSD and may be taken advantage of during therapeutic intervention.

## Introduction

Headache is a common symptom of several neurologic disorders (Pitts et al., 2008; Tabatabai and Swadron, 2016). Disorders presenting with headache can be classified as either primary or secondary headache disorders depending on their underlying cause [Headache Classification Committee of the International Headache Society (IHS), 2013]. One mechanism implicated in both primary and secondary disorder production is cortical spreading depression (CSD; Olesen et al., 1990; Hadjikhani et al., 2001; Bolay et al., 2002; Dreier et al., 2006; Hartings et al., 2009; Zhang et al., 2010; Lauritzen et al., 2011; Woitzik et al., 2013; Chen and Ayata, 2016). CSD is described as a wave of self-propagating depolarization followed by hyperpolarization across the cortex (Leo 1944; Leao 1947; Somjen, 2001). Induced CSD events can lead to potentiation of excitotoxicity, production of CNS lesions, and changes in cerebral blood flow (Chen and Ayata, 2016). Additionally, CSD has been suggested to trigger activation and persistent sensitization of meningeal nociceptors, potentially contributing to the production of pain in headache disorders (Zhang et al., 2010; Zhao and Levy, 2016). CSD has been shown to elicit the release of pronociceptive and vasoactive substances including calcitonin gene-related protein (CGRP), glutamate, ATP, nitric oxide, potassium, and hydrogen ions in the cortex (Brennan et al., 2007; Russo, 2015). Some of these substances independently are reported to influence blood-brain barrier (BBB) permeability (Diler et al., 2007; Asghar, 2012; Eftekhari et al., 2015; Wang et al., 2015).

Previous studies have provided evidence that BBB permeability is altered following CSD. In addition to contributing to deleterious events in the CNS, altered BBB permeability has the potential to impact drug delivery to the CNS during and following CSD (Gursoy-Ozdemir et al., 2004; Olah et al., 2013; Friedman and Kaufer, 2015). The BBB, at the level of the capillary endothelial cells, is the main CNS component involved in regulating the passage of substances from the circulating blood to the brain and vice versa (Abbott et al., 2010). The BBB is comprised of specialized endothelial cells joined together to form tight junctions (TJs) with high transendothelial cell resistance (TEER; Abbott et al., 2010). These TJs are comprised of TJ proteins, such as occludin and the claudins, which are anchored to the actin cytoskeleton by the zona occludens (ZO) linker proteins. Importantly, these interactions are dynamic, and therefore change in response to surrounding cues (Abbott et al., 2010). This dynamic nature of the BBB is regulated in part by the neurovascular unit (NVU). The NVU is comprised of numerous CNS cell types including endothelial cells, astrocytes, other glial cells, neurons, and pericytes (Hawkins and Davis, 2005). Based on the ability of the BBB to react to changes in CNS activity, it is highly likely that changes in BBB permeability occur in CNS disorders such as headache and pain.

Some preclinical work has suggested that BBB permeability is increased in response to CSD or headache pain (Gursoy-Ozdemir et al., 2004). Separately, it has also been shown that KCl-induced CSD elicits nociceptive behaviors in rats; however, BBB permeability was not measured (Fioravanti et al., 2011). Additionally, examination of BBB permeability in a dural inflammation model of headache showed a site-specific increase in BBB permeability in the trigeminal nucleus caudalis (TNC), a brain region involved in transmission of nociceptive stimuli from the trigeminal afferents (Fried et al., 2017). A few clinical studies have also attempted to study BBB changes in response to primary headache disorders such as migraine but have produced conflicting results. While one study on spontaneous migraine with aura observed an increase in matrix metalloproteinase-9 (MMP-9), indicative of a BBB breach (Leira et al., 2007), another study in migraine with aura found increased perfusion at the brainstem only and no permeability changes at the cortex (Hougaard et al., 2017). No clinical or preclinical study to date, however, has successfully examined the temporal relationship between headache pain, CSD, paracellular BBB integrity and/or leak in the context of drug delivery.

In the current study, we hypothesized that CSD induced by a single KCl injection would increase paracellular CNS uptake of small molecules at discrete times during headache induction. Using a combinatorial approach of electrophysiology, behavioral measures, and *in situ* brain perfusion, we found that cortical KCl injection induced CSD and temporally increased paracellular BBB leakiness to radiolabeled sucrose in the cortex, but not in the brainstem. We also observed an increase in BBB permeability to sumatriptan in both the cortex as well as the brainstem, indicating the potential for changes in active transport activity.

## Materials and Methods

### Animals

Female Sprague Dawley rats (200–250 g) were purchased from Envigo and housed in a climate-controlled room on a regular 12/12 h light/dark cycle with lights on at 7 A.M. with food and water available *ad libitum* in the vivarium five floors below the lab. All procedures were performed during the light phase and according to the policies and recommendations of the International Association for the Study of Pain, the National Institutes of Health (NIH) guidelines for laboratory animals, and with approval from the Institutional Animal Care and Use Committee of the University of Arizona. For Number of Animals Consistent with NIH policy (NOT-OD-15-102) on relevant biological variables (e.g., strain, sex, and age) experiments were randomized to blinded treatment (topiramate, sumatriptan, or vehicle), injury (KCl or aCSF), and control groups, giving 80% power to detect a treatment effect size of 20% compared to a baseline response of 5% at a significance level of 0.05. Numbers required to achieve statistical power for each experiment were determined by G*Power 3.1. Female rats were used as headache disorders affect females to males at a nearly 3:1 ratio (Bolay et al., 2011; Stucky et al., 2011; Misra et al., 2013; Chai et al., 2014; Faria et al., 2015).

### Vaginal smears

Estrous cycles of intact female rats were monitored by daily vaginal smears. The vaginal smears were interpreted as described by Goldman et al. (2007). Briefly, vaginal openings were flushed with 200 µl of sterile saline. Fresh samples were evaluated for cytology at the same time daily for at least 8 d using a Zeiss Axioskop 40 (10×/0.3 numerical aperture EC Plan-Neofluar objective, Carl Zeiss Microscopy). Cortical injections were given during the diestrous phase of the estrous cycle. CSD events, pain behaviors, and BBB permeability were also assessed during the diestrous phase; except in the case of multiple day assessments, where rats continued to progress through the estrous cycle.

### Drugs and reagents

Ketamine/xylazine was purchased from Sigma-Aldrich. ^14^C-sucrose and optiphase supermix scintillation cocktail were purchased from PerkinElmer. ^3^H-sumatriptan was purchased from American Radiolabeled Chemicals Inc. Sumatriptan was generously donated by the Porreca Lab (University of Arizona; source: Abmole Bioscience). Topiramate was purchased from Cayman Chemical. TS-2 tissue solubilizer was purchased from Research Products International. EDTA-free complete protease inhibitors were purchased from Roche. The Coomassie Plus Better Bradford Assay kit was purchased from Thermo Scientific. XT sample buffer, XT reducing agent, Precision Plus dual color prestained molecular weight markers, and TGX criterion gels were purchased from Bio-Rad. Primary antibodies used for Western blotting (WB) include the following: claudin-5 [Invitrogen (Thermo Fisher), catalog #4C3C2, 1:500], occludin [Invitrogen (Thermo Fisher), catalog #OC-3F10, 1:1000], and α-tubulin (Cell Signaling, catalog #DM1A, 1:5000). Secondary antibodies for WB were purchased from Cell Signaling Technology and used at a 1:20,000 dilution. All other chemicals were purchased from Sigma-Aldrich.

### Dural cannulation

Anesthesia was induced with intraperitoneal 80:12 mg/kg ketamine:xylazine. Rats were placed in a stereotactic frame (Stoelting Co.), and a 1.5- to 2-cm incision was made to expose the skull. A 0.66- to 1-mm hole (Pinprick/KCl: -6 mm A/P, -3 mm M/L from bregma) was made with a hand drill (DH-0 Pin Vise; Plastics One) to carefully expose, but not damage, the dura. A guide cannula (0.5 mm from top of skull, 22 GA, #C313G; Plastics One) was inserted into the hole and sealed into place with glue. Two additional 1mm holes were made caudal to the cannula to receive stainless-steel screws (#MPX-080-3F-1M; Small Parts), and dental acrylic was used to fix the cannula to the screws. A dummy cannula (#C313DC; Plastics One) was inserted to ensure patency of the guide cannula. Rats were housed individually and allowed 6–8 d to recover. Cannula placement and dural integrity was confirmed postmortem.

### Cortical injections

Cortical injections were performed by employing a Hamilton injector (30 GA, #80308 701 SN, Hamilton Company) customized to project 1.0 mm into the brain. The injector was inserted through the guide cannula to deliver a focal injection of 0.5 µl of 1 M KCl or artificial CSF (aCSF) into the cerebral cortex. aCSF was comprised of 145 mM NaCl, 2.7 mM KCl, 1 mM MgCl_2_, 1.2 mM CaCl_2_, and 2 mM Na_2_HPO_4_ (pH 7.4), and the solution was passed through a 0.2-µm syringe filter before injection.

### Precortical injection treatments

Topiramate (40 mg/kg in suspension) or vehicle (saline) was injected intraperitoneally (IP) 30 min before cortical injection of either KCl or aCSF. Permeability of the BBB was assessed 1.5 h postcortical injection.

### Implantation of recording electrodes

Silver chloride (AgCl) electrodes were prepared by flaming 0.25 mm Ag wire (A-M Systems, Inc.) into spherical tips (1 mm in diameter) and coating the tips with Cl^-^. Rats were anaesthetized with ketamine/xylazine (as above) then fixed to a stereotaxic frame (Stoelting). Three burr holes were drilled through the skull using a manual drill to allow placement of the AgCl recording electrodes. The frontal and parietal lead electrodes were placed from bregma: 2 mm lateral and 1.5 mm anterior, 2 mm lateral and -2.5 mm posterior to bregma, respectively; the reference electrode was placed -7.5 mm A/P, 2 mm M/L. Two screws (#MPX-080-3F-1M, Small Parts Inc.) were fastened into the skull without going through it. The four electrodes were soldered into a multi-pin connector (Continental Connector), and the apparatus was fixed into place using dental cement. Animals were housed individually and allowed 2–3 d to recover to ensure electrode integrity for recordings.

### Electrophysiological recordings

Forty-eight hours after dural electrode implantation, rats were placed in a recording chamber (40 cm long × 49 cm wide × 37 cm high) and the multi-pin connector attached to an electro-cannular swivel (#CAY-675-6 commutator, Airflyte) mounted in the ceiling of the chamber. The swivel allowed rats to move freely about the chamber during the recording period. Animals were allowed to habituate to the chamber for 2 h to permit electrical recordings to stabilize. Only those rats with stable electrical recordings were included in experimental groups. Signals leading to separate DC and AC amplifiers (Grass Model 15 amplifier system, 15A12 DC and 15A54 AC amplifiers, Astro-Med Inc.) through insulated cables and were collected with EEG recording analysis software Gamma v.4.9 (Astro-Med, Inc.). The recording data were reviewed with Gamma Reviewer (Astro-Med, Inc.) and analyzed for SD events offline. SD was defined by significant reductions in both the power and amplitude of the EEG tracings and was only considered as an event when (1) the AC current was reduced by half; (2) the DC current exhibited a downward shift by a minimum of 1mV; and (3) the duration was a minimum of 30 s.

### Behavioral assessment

Periorbital allodynia was evaluated before and at 0.5, 1, 1.5, 3, 6, 24–72 h after cortical injection. Rats were grouped based on their postsurgical baseline to ensure equivalent preinjection thresholds, any rats exhibiting excessive postsurgical allodynia (threshold <4 g) were removed from the study. Rats were acclimated to the testing box 90 min before evaluation of periorbital mechanical allodynia with calibrated von Frey filaments (Stoelting). Calibrated von Frey filaments were applied perpendicularly to the midline of the forehead at the level of the eyes until the filament was slightly bent while held for 5 s. A positive response was indicated by a sharp withdrawal of the head or vocalizing. The withdrawal threshold was determined using a modified version of the Dixon up-down method, where the response pattern was used to obtain the threshold as reported by Chaplan et al. (1994). Rearing events were assessed for 5-min intervals before and at 0.5, 1, 1.5, 3, 6, 24–72 h after cortical injection. Vertical rears were counted each time a rat stood on both hind paws without grooming.

### *In situ* brain perfusion

Briefly, rats were anesthetized with ketamine/xylazine (as described above) and heparinized (10,000 U/kg, i.p.). Body temperature was maintained at 37°C using a heating pad. The common carotid arteries were bi-laterally cannulated and connected to a perfusion circuit. The perfusate was an erythrocyte-free modified mammalian Ringer’s solution: 117 mM NaCl, 4.7 mM KCl, 0.8 mM MgSO_4_, 1.2 mM KH_2_PO_4_, 2.5 mM CaCl_2_, 10 mM D-glucose, 3.9% (w/v) dextran (MW 60,000), and 1.0 g/l bovine serum albumin (Type IV), pH 7.4, warmed to 37°C and oxygenated with 95% O_2_/5% CO_2_, Evan’s blue dye (55 mg/l) was added to the perfusate to serve as a visual marker of BBB integrity. Perfusion pressure and flow rate were maintained at 95–105 mmHg and 3.1 ml/min, respectively. Both jugular veins were severed to allow for drainage of the perfusate. Using a slow-drive syringe pump (0.5 ml/min per hemisphere; Harvard Apparatus), ^14^C-sucrose (0.5 μCi/ml) or ^3^H-sumatriptan (0.25 μCi/ml) was added to the inflowing perfusate for 10 min followed by a 2-min washout period in which non-radioactive Ringer’s solution was perfused to clear vascular radioactivity. Following perfusion, the rat was decapitated and the brain removed. The meninges and choroid plexus were removed, cerebral hemispheres were sectioned and the brain was divided and placed into preweighed vials. TS2 tissue solubilizer (1 ml) was added to each tissue sample, and the samples were solubilized for 2 d at room temperature. To eliminate chemiluminescence, 100 μl of 30% glacial acetic acid was added, along with 1.5 ml Optiphase SuperMix liquid scintillation cocktail (PerkinElmer). Perfusion media was also sampled and placed in triplicate 100-μl aliquots in scintillation vials and processed in the same manner as the tissue samples. All samples were then measured for disintegrations per minute (dpm; 1450 LSC and Luminescence Counter; PerkinElmer). The ratio of the concentration of ^14^C-sucrose or ^3^H-sumatriptan in tissue (C_brain_; in dpm/g) was compared with perfusate (C_perfusate_; in dpm/ml) and expressed as a percentage ratio (RBR) R_brain_ = (C_brain_/C_perfusate_) × 100%.

### Cortical tissue collection

Rats were anesthetized with ketamine/xylazine mix as described above, decapitated, and the cortices placed in ice-cold collection buffer (136.9 mM NaCl, 2.7 mM KCl, 1mM CaCl_2_, 1.5 mM KH_2_PO_4_, 8.1 mM Na_2_HPO_4_, 0.5 mM MgCl_2_, 5 mM glucose, and 1 mM sodium pyruvate; pH 7.4) supplemented with Roche EDTA-free Complete Protease Inhibitor cocktail, Sigma protease inhibitor cocktail and 2 mM phenylmethylsulfonyl fluoride. All subsequent steps were performed on ice or at 4°C. Choroid plexus and meninges were removed, three rat brains/treatment were pooled and homogenized in 20-ml collection buffer using a Potter-Elvehjem homogenizer followed by eight strokes in a glass dounce homogenizer by hand. Samples were stored at −80°C until use.

### WB

Tissue samples were prepared with inhibitors as described above. Samples were incubated for 10 min at 70°C and centrifuged for 5 min at 13,000 × *g* at 21°C to separate any solid particles before gel electrophoresis; 10 µg of total proteins from the tissue supernatant were loaded on TGX precast gels and separated by SDS-PAGE and then transferred to polyvinylidene difluoride membranes (Imobilon, Millipore) and blocked at room temperature for 1 h. Primary antibodies and dilutions are given above. All antibodies were diluted in 5% BSA in tris-buffered saline with Tween 20 (TBST). Immunoblots were revealed by enhanced chemiluminescence (WBKLS0500, Millipore) and imaged on photographic film. To visualize multiple bands on the same blot, blots were stripped with Restore Western Blot Stripping buffer (Pierce, Thermo Fisher). Films were scanned, digitized, and quantified using Un-Scan-It gel version 6.1 scanning software (Silk Scientific Inc.). Protein expression of TJ proteins were corrected for expression of the loading control α-tubulin to normalize for protein expression.

### Statistical analysis

GraphPad Prism 7.0 software (GraphPad Software) was used for statistical analysis. Unless otherwise stated, the data were expressed as mean ± SEM. Periorbital allodynia measurements and *in situ* perfusion time course measurements were assessed using a repeated measure two-way ANOVA to analyze differences between treatment groups over time with a Bonferroni test applied *post hoc*. In rearing experiments, data were expressed as Area Under the Curve (AUC) ± SEM for rears performed for the duration of the time course. Differences between groups were assessed using an unpaired *t* test with Welch’s correction. Molecular studies were compared by one-way ANOVA. Single timepoint *in situ* perfusion results were compared by one-way ANOVA and an unpaired *t* test with Welch’s correction to determine statistical significance. When *p* values were ≤0.05, they were accepted as statistically significant.

## Results

### Cortical injection of KCl, but not aCSF, produces nociceptive behaviors

CSD has been implicated in headache production in both primary and secondary headache disorders. Studies examining the influence of CSD on nociceptive behaviors and BBB changes have used male rats to assess these parameters (Gursoy-Ozdemir et al., 2004; Fioravanti et al., 2011; Olah et al., 2013). It is known, however, that migraine and many of the underlying disorders leading to secondary headache production afflict women more than men (Hu et al., 1999; Haast et al., 2012). Based on these factors we chose to conduct our studies in intact female rats. As a first step to establish this model in female rats, we measured postsurgical facial allodynia following surgical cannula implantation. Postsurgical facial withdrawal thresholds were 5.74 ± 0.47 g compared to facial withdrawal thresholds of 8 g (maximum measured) presurgery. This difference remained unresolved up to 12 d after surgery (data not shown).

**Figure 1.**
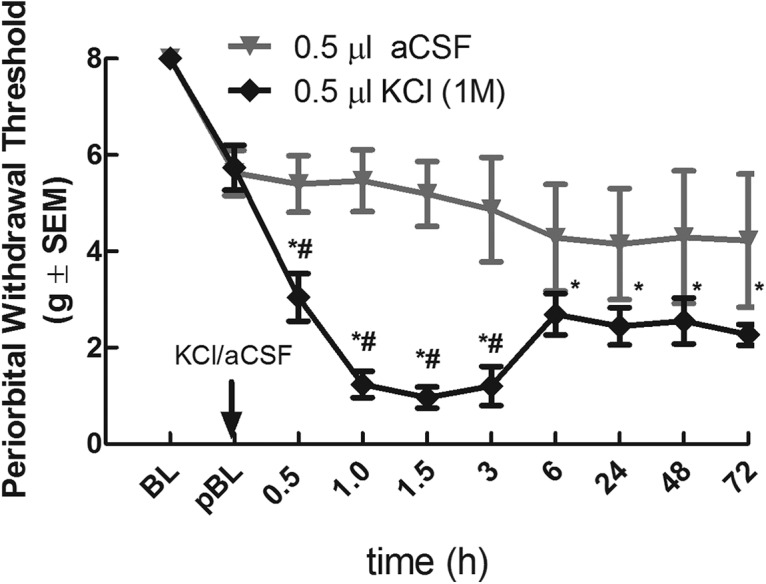
Cortical injection of 1M KCl induces periorbital allodynia. ***A***, Prior to surgery rats do not exhibit significant periorbital allodynia (BL). Cortical injection of KCl (1 M, 0.5 µl), but not aCSF (0.5 µl) significantly decreased facial withdrawal thresholds beginning 30 minutes post injection which improved by 6 hours but did not return to pre-injection baseline thresholds during the 72 hour timecourse. * Denotes significant (*p* < 0.05) differences from post-surgery baseline (pBL). # denotes significant (*p* < 0.05) differences from aCSF injected group (KCl *N* = 21, aCSF *N* = 16).

To assess the ability of CSD to induce headache-like nociceptive behaviors, rats received a cortical injection of 1 M KCl to disrupt the extracellular ion equilibrium and the development of CSD was monitored (Fioravanti et al., 2011). All rats injected with cortical KCl experienced up to 2 CSD events within the first 2 h of recording postcortical injection. Cortical aCSF injection, on the other hand, did not produce any observed CSD events (Table 1). Rats receiving a cortical KCl (*N* = 21) injection exhibited significantly reduced periorbital withdrawal thresholds within 0.5 h, which peaked between 1 and 3 h. Facial withdrawal thresholds recovered significantly within 6 h, but remained significantly lower than preinjection baselines for the remainder of the 72-h time course (KCl withdrawal thresholds at 0.5 h 3.043 ± 0.49, *p* = 0.0003^a^, 1 h 1.23 ± 0.27, *p* < 0.0001^a^, 1.5 h 0.96 ± 0.22, *p* < 0.0001^a^, 3 h 1.21 ± 0.41, *p* < 0.0001^a^, 6 h 2.70 ± 0.41, *p* = 0.0014^a^, 24 h 2.44 ± 0.39, *p* = 0.0003^a^, 48 h 2.55 ± 0.48, *p* = 0.0062^a^, and 72 h 2.27 ± 0.21, *p* = 0.004^a^). A separate group of animals receiving a 0.5-μl cortical injection of aCSF (*N* = 16) did not exhibit significantly decreased facial withdrawal thresholds from baseline at any of the examined time points (Fig. 1) suggesting that head nociception was not induced. Non-evoked, or pain-suppressed behavior, was also assessed in response to CSD. Previous studies have shown that rearing behaviors are suppressed in response to facial allodynia (Sandweiss et al., 2017; Zhang et al., 2017). The ability of cortical KCl to depress rearing behavior was assessed throughout the 72-h behavioral time course. Throughout the behavioral time course, total rearing events were significantly decreased compared to rats injected with aCSF (AUC aCSF 850.5 ± 83.04, *N* = 10, AUC KCl 302.5 ± 54.45, *N* = 12, *p* = 0.0002^b^). These data support the validity of cortical KCl injection in awake, freely moving female rats as a model of CSD-induced episodic headache.

**Table 1.** Summary of CSD parameters from rats given a cortical injection of either KCl (*n* = 3) or aCSF (*n* = 3)

Group	Number of CSD events	EEG percent decrease	Frontal CSD amplitude decrease (mv)	Parietal CSD amplitude decrease (mV)	Duration (s)
KCl (1 M)	9	64.39 ± 3.16	2.56 ± 0.53	2.11 ± 0.47	59.79 ± 5.84
aCSF	0	N/A	N/A	N/A	N/A

Rats given a cortical KCl injection experienced at least one CSD event within the recording period. Rats injected cortically with aCSF did not experience CSD. CSD was marked by a significant reduction in both the power and amplitude of the EEG tracings and was only considered as an event when (1) the AC current was reduced by half; (2) the DC current exhibited a downward shift by a minimum of 1 mV; and (3) the duration was a minimum of 30 s.

### BBB permeability is increased following cortical KCl injection at time points corresponding to facial allodynia incidence

**Figure 2.**
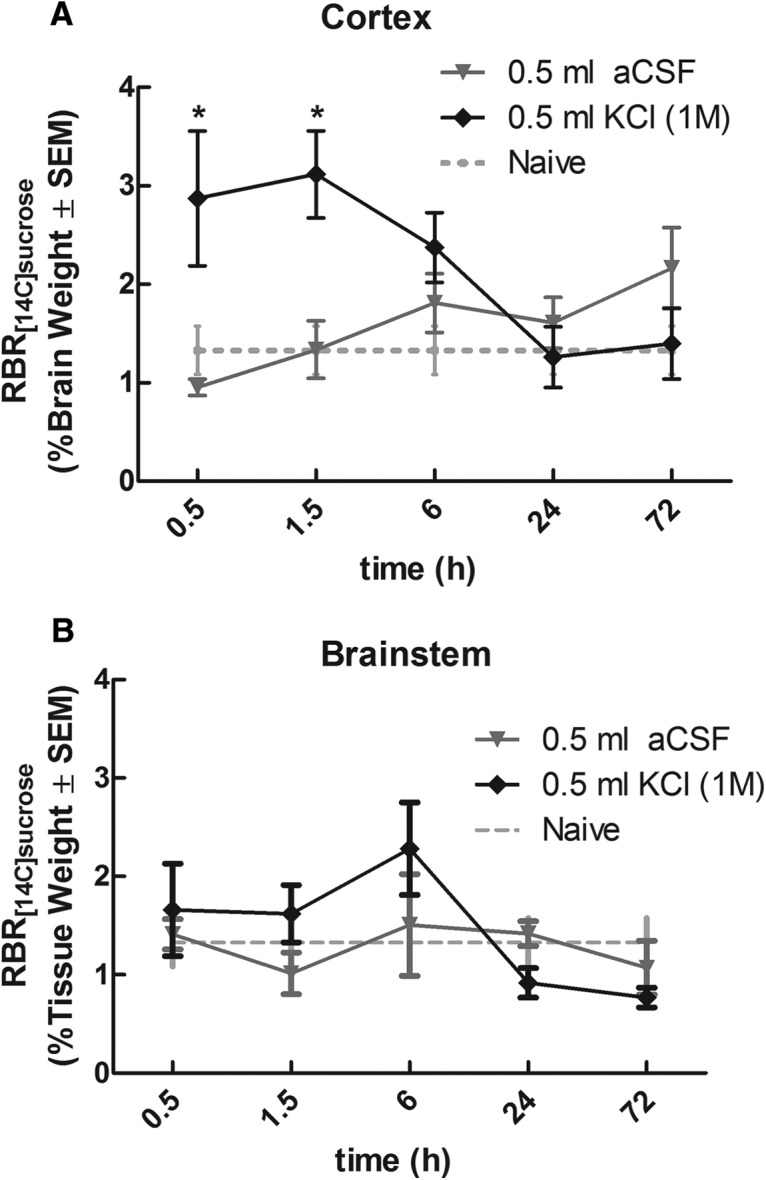
Cortical KCl injection increased BBB permeability to ^14^C-sucrose in the cortex but not brainstem as assessed with *In situ* brain perfusion. ***A***, Cortical injection of KCl (0.5 μl, 1M) induced an increase in cortical BBB permeability to ^14^C-sucrose at 30 minutes post injection. Permeability returned to naïve levels (indicated by dashed line) within 6 hours. ***B***, Injection of KCl (0.5 μl, 1M) into the cortex did not produce significant changes in brainstem permeability to ^14^C-sucrose. Naïve values for ^14^C-sucrose are denoted by the dashed line. The amount of radioactivity in the cortex and brainstem versus the perfusate was expressed as relative brain uptake or RBR%. * Denotes significant (*p* < 0.05) differences compared to naïve uptake (*N* = 8–10/group/time-point).

The ability of CSD to induce changes in BBB integrity has been demonstrated in a handful of studies in anesthetized animals (Gursoy-Ozdemir et al., 2004; Olah et al., 2013). No studies to date, however, have assessed BBB integrity or permeability in relation to behavioral changes following cortical KCl injection in awake, freely moving rats. Following determination of facial withdrawal thresholds, cortical BBB permeability was assessed using the *in situ* brain perfusion technique. ^14^C-sucrose (MW = 342.30 g/mol) was used to evaluate paracellular permeability (leak) at the BBB due to its relative inability to cross the BBB under non-stressed/normal conditions and due to the lack of any known active transport to the brain. Compared to naive rats (RBR 1.68 ± 0.35, *N* = 8), cortical KCl injection increased BBB permeability of ^14^C-sucrose in the cortex within 0.5 h (RBR 4.24 ± 0.17 *N* = 7, *p* = 0.0217^a^; Fig. 2*A*). Permeability returned to naive levels within 6 h post-KCl injection (RBR 3.12 ± 0.13, *N* = 10, *p* = 0.0037^a^ at 1.5 h and 2.37 ± 0.12, *N* = 8, *p* = 0.0680^a^ at 6 h; Fig. 2*A*). This time course of increased cortical BBB permeability corresponds to the duration of periorbital allodynia seen during behavioral assessment following cortical KCl injection. No change in BBB permeability was seen with cortical aCSF injection compared to naive rats (Fig. 2*A*). Due to the importance of the brainstem in the transmission of nociceptive signals related to headache production, BBB permeability was also assessed in the brainstem of rats receiving a cortical injection of KCl. Rats injected with either cortical KCl or aCSF showed no change in BBB permeability to ^14^C-sucrose in the brainstem compared with naive rats (naive RBR 1.632 ± 0.356, *N* = 9; Fig. 2*B*). These data suggest the ability of KCl injected cortically in awake, freely moving rats to induce significant changes in BBB permeability in the cortex, but not brainstem, at times corresponding with decreased facial withdrawal thresholds.

### Cortical KCl injection does not alter total expression of TJ proteins occludin or claudin-5

**Figure 3.**
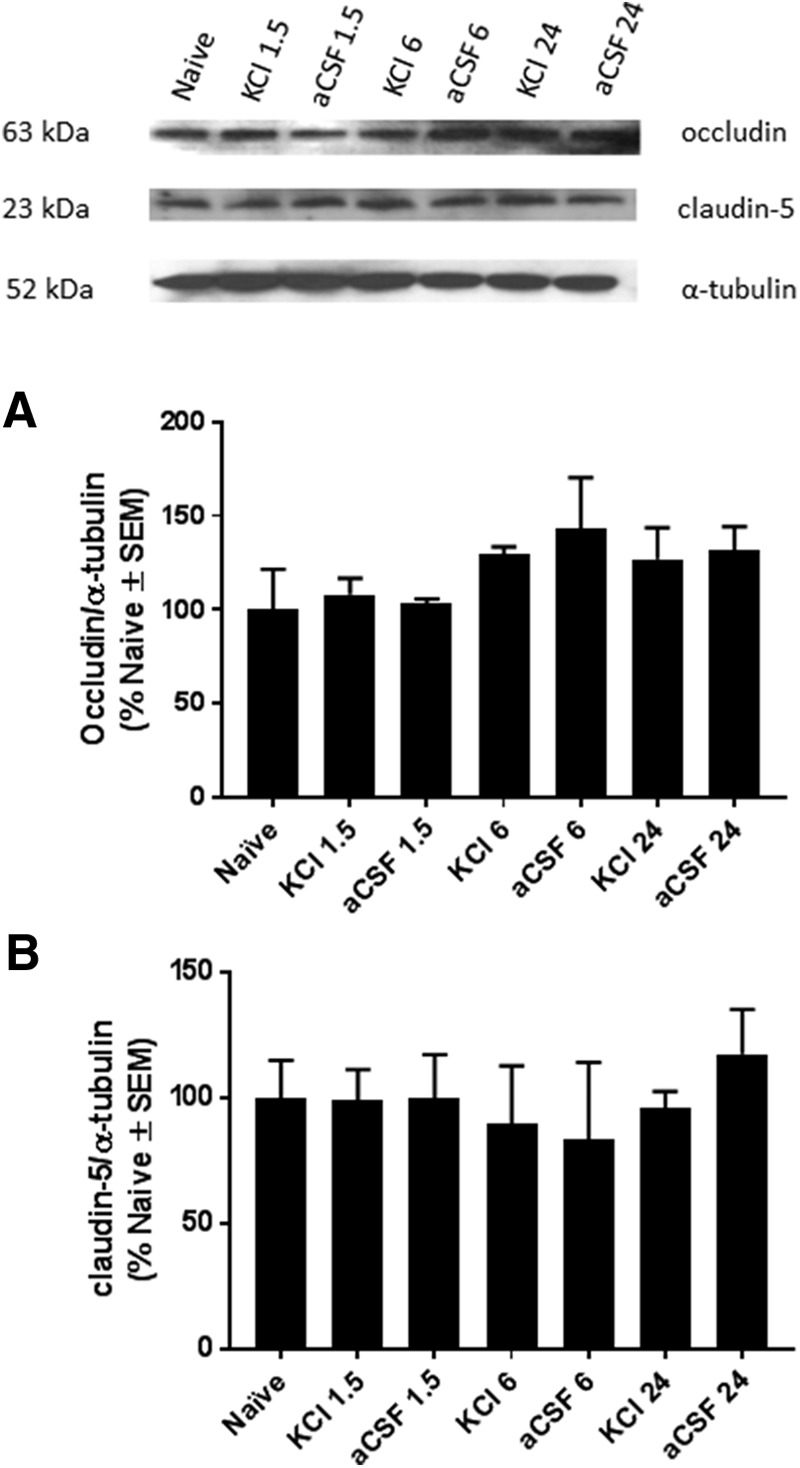
Protein expression of occludin (***A***) and claudin-5 (***B***) in cortical samples. The Western blot analysis of whole cortex samples showed statistically insignificant fluctuations in the total expression of tight junction proteins compared to naïve rats following either cortical KCl or aCSF injection (*N* = 3 pools of 3 rats).

Many studies have shown decreases in TJ protein expression in conjunction with changes in BBB permeability. To determine whether cortical KCl-induced BBB leak corresponded to changes in TJ protein expression, we assessed expression of TJ proteins occludin and claudin-5 following cortical KCl treatment. In samples from whole cortex, expression of claudin-5 was statistically unchanged compared to naive rats following either cortical KCl or aCSF injection (*N* = 3 pools of three rats^c^; Fig. 3*A*). Samples from whole cortex also revealed no change in occludin expression in response to either cortical KCl or aCSF (*N* = 3 pools of three rats^c^; Fig. 3*B*). This data suggests that other changes in BBB structure (i.e., TJ protein localization) are likely involved in eliciting the permeability changes seen in response to cortical KCl-induced CSD.

### Cortical KCl injection increases BBB permeability to radiolabeled sumatriptan

**Figure 4.**
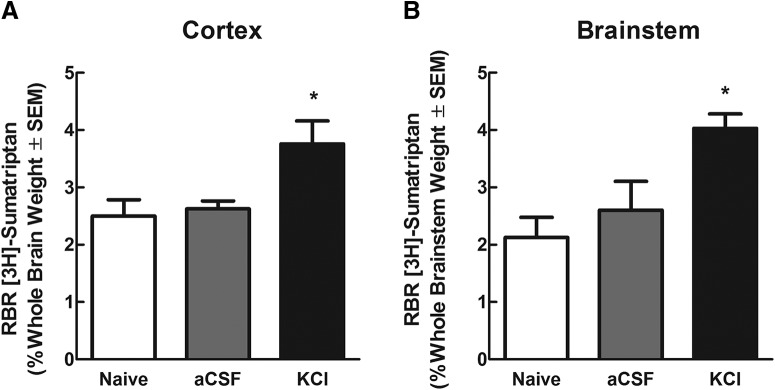
Cortical KCl injection increased both cortical and brainstem BBB permeability to ^3^H-sumatriptan as assessed by In situ brain perfusion. ***A***, Leakiness of the BBB to ^3^H-sumatriptan was observed in response to cortical KCl injection compared to naïve values. No significant difference was observed between naïve rats and those given a cortical aCSF injection. ***B***, Brainstem permeability to ^3^H-sumatriptan was also increased in response to cortical KCl injection 1.5 hours after injection. The amount of radioactivity in the cortex and brainstem versus the perfusate was expressed as relative brain uptake or RBR%. * Denotes statistically significant (*p* < 0.05) differences compared to naïve uptake. (Naïve *N* = 11, aCSF *N* = 7, KCl *N* = 8).

Sumatriptan is a drug used as an abortive agent for the treatment of migraine. Additionally, sumatriptan has been reported to improve secondary headaches resulting from several conditions including meningitis and subarachnoid hemorrhage (Ferrari et al., 2002; Ahn and Basbaum, 2005; Girdley and Rifkin, 2012). Studies have shown minimal crossing of sumatriptan through the BBB, suggesting it works primarily in the periphery (Ahn and Basbaum, 2005); however, some experiments have demonstrated the ability of triptans such as sumatriptan to act as direct inhibitors of second order neurons in the TNC in the brainstem (Cumberbatch et al., 1997; Goadsby et al., 2001). Permeability to ^3^H-sumatriptan (mw = 295.402 g/mol) was determined 1.5 h after cortical KCl injection. The 1.5-h time point was chosen for this and subsequent experiments based on behavioral data showing peak facial allodynia 1.5 h postinjection as well as significant BBB leak (Table 2). Uptake of ^3^H-sumatriptan was initially assessed in naive rats and showed minimal uptake in both the cortex and brainstem; consistent with previous studies (Table 2). Consistent with results seen using ^14^C-sucrose, uptake of ^3^H-sumatriptan was significantly increased in the cortex 1.5 h post-KCl injection (naive RBR 2.50 ± 0.29 *N* = 11, KCl RBR 3.756 ± 0.41 *N* = 8, *p* = 0.0182^b,c^; Fig. 4*A*). In contrast to the results using ^14^C-sucrose, uptake of ^3^H-sumatriptan was also robustly increased in the brainstem 1.5 h postinjection (naive RBR 2.12 ± 0.35 *N* = 11, KCl RBR 4.03 ± 0.25 *N* = 8, *p* = 0.0004^b,c^; Fig. 4*B*). As with studies using ^14^C-sucrose, rats injected with cortical aCSF had no significant change in BBB permeability compared to naive rats (Fig. 4*A*,*B*). These data suggest the ability of sumatriptan, to reach headache relevant CNS via active transport in brainstem areas such as the TNC, where it may exert additional therapeutic actions.

**Table 2.** Summary of values from significant effects of cortical KCl at the 1.5-h time point compared to naive values

Treatment group	Parameter	Value
Naive	Cortex, [^14^C]-sucrose RBR	1.68 ± 0.32% weight
Naive	Cortex, [^3^H] sumatriptan RBR	2.50 ± 0.29% weight
Naive	Brainstem, [^3^H] sumatriptan RBR	2.13 ± 0.35% weight
Postsurgical baseline	Facial withdrawal threshold	5.74 ± 0.47 g
Cortical KCl (1 M, 0.5 µl)	Cortex, [^14^C] sucrose RBR	3.12 ± 0.13% weight
Cortical KCl (1 M, 0.5 µl)	Cortex, [^3^H] sumatriptan RBR	3.76 ± 0.41% weight
Cortical KCl (1 M, 0.5 µl)	Brainstem, [^3^H] sumatriptan RBR	4.03 ± 0.25% weight
Cortical KCl (1 M, 0.5 µl)	Facial withdrawal threshold	0.96 ± 0.22 g

For reference, statistically significant BBB leak and facial allodynia were also observed in Figs. 1, 2.

### Acute topiramate does not prevent increased BBB permeability in response to cortical KCl injection

**Figure 5.**
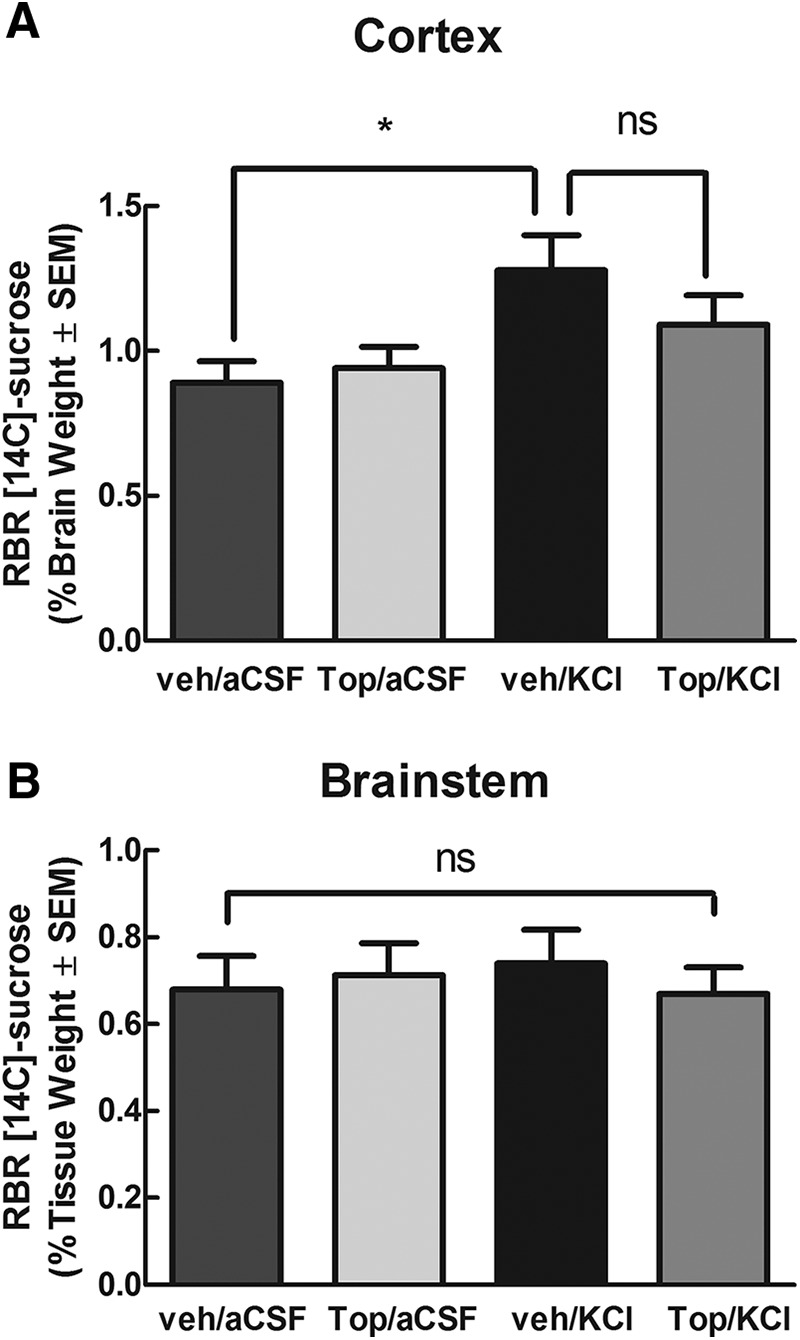
Pre-treatment with topiramate 30 minutes prior to CSD induction with cortical KCl did not prevent KCl induced BBB leakiness to ^14^C-sucrose as assessed by In situ brain perfusion. ***A***, Cortical KCl injection increased ^14^C-sucrose uptake in the cortex. This increase in leak was not changed by pre-treatment with the anti-epileptic and headache prophylactic drug topiramate. ***B***, No significant difference were observed in brainstem permeability to ^14^C-sucrose in response to either KCl or topiramate. Topiramate did not alter BBB permeability in either the cortex or brainstem in control, vehicle/aCSF treated rats. The amount of radioactivity in the cortex and brainstem versus the perfusate was expressed as relative brain uptake or RBR%. NS denotes no significant difference between groups. * Denotes significant (*p* = 0.0279) differences compared to vehicle/aCSF treated rats (vehicle/aCSF *N* = 21, topiramate/aCSF *N* = 17, vehicle/KCl *N* = 22, topiramate KCl *N* = 23).

Topiramate is an anti-epileptic drug which has also been employed for the prevention of primary headache disorders such as migraine (Akerman and Goadsby, 2005). Studies have shown that topiramate can block CSD production in response to cortical KCl application (Akerman and Goadsby, 2005; Unekawa et al., 2012). To assess whether a single injection of topiramate, as a CSD preventative, blocked observed cortical BBB leakiness in response to KCl, topiramate or vehicle (saline) was given 30 min before cortical KCl injection. Increased BBB permeability to ^14^C-sucrose in response to cortical KCl injection was not prevented by pretreatment with topiramate (topiramate +KCl RBR 1.091 ± 0.4856 *N* = 23, vehicle + KCl RBR 1.314 ± 0.5571 *N* = 22^b,c^; Fig. 5*A*). In rats given cortical aCSF injections, topiramate produced no significant changes in ^14^C-sucrose uptake compared to those treated with vehicle, suggesting that topiramate alone does not produce changes in BBB permeability (topiramate +aCSF RBR 0.9405 ± 0.3069 *N* = 17, vehicle + aCSF RBR 0.8902 ± 0.3411 *N* = 21^b,c^; Fig. 5*A*). Additionally, in agreement with permeability data shown in Figure 2, no significant changes were seen in ^14^C-sucrose uptake within the brainstem in any treatment group (Fig. 5*B*). Importantly, pretreatment with a single dose of topiramate did not significantly prevent KCl-induced periorbital allodynia (data not shown). In prior experiments, KCl-induced periorbital allodynia and BBB leak occurred on a similar time course. These data suggest that pretreatment with a single dose of topiramate is not sufficient to prevent cortical KCl-induced BBB leakiness and that the mechanism of barrier leak may be multifaceted, related to both nociceptive transmission as well as other neurovascular mechanisms (Table 3).

**Table 3.** Summary of Statistical Analyses used. Differences were considered significant at *p* < 0.05

Data structure	Type of test	Power
a. Normal distribution	Two-way ANOVA	*p* < 0.05
b. Normal distribution	Unpaired *t* test with Welch’s correction	*p* < 0.05
c. Normal distribution	One-way ANOVA	*p* < 0.05

## Discussion

**Figure 6.**
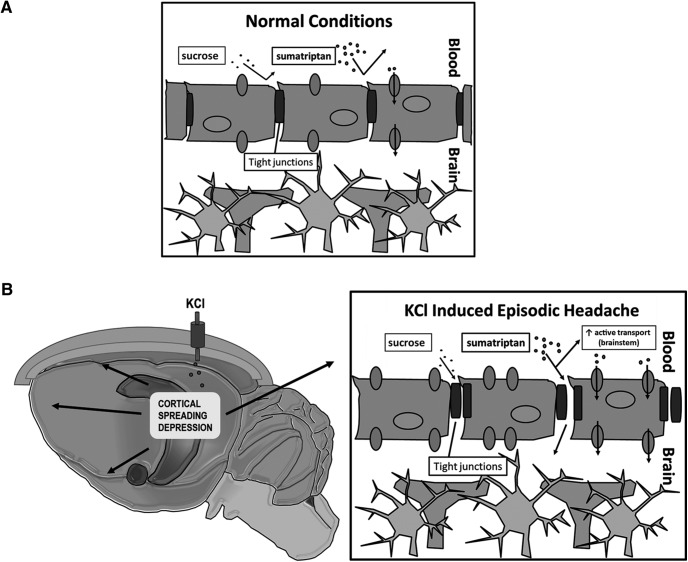
Schematic of effects of KCl induced spreading depression on BBB permeability in microvasculature. ***A***, Under normal conditions, both sucrose and sumatriptan have minimal penetration into the CNS. ***B***, Following KCl induced CSD, both sucrose and sumatriptan have increased entry into the cortex. This increased entry can be explained via an increase in paracellular leak of the BBB, either through changes in tight junction function or localization. Additionally, increased uptake of sumatriptan, but not sucrose, was seen in the brainstem. This increased uptake in the brainstem may be due to increased active transport.

In conditions such as TBI and ischemic stroke, the integrity of the BBB is altered following injury (Prakash and Carmichael, 2015), although this loss of integrity is most often associated with trauma within the CNS rather than with CSD or headache. Additionally, in studies examining primary headache disorders, where no obvious CNS trauma exists, CSD and increased BBB permeability have been shown to occur in some instances (i.e., migraine with aura; Leira et al., 2007; Charles and Baca, 2013; Fried et al., 2017). The data presented here represent the first study examining, concurrently, the temporal occurrence of CSD, nociceptive behaviors, and BBB leak in a CSD-induced model of episodic headache. Our data suggest that KCl-induced CSD in awake, freely moving female rats increases cortical, but not brainstem, BBB paracellular permeability to ^14^C-sucrose. This permeability change occurs in parallel to CSD events and periorbital allodynia. Importantly, this model was designed without induction of other CNS injuries such as ischemia or trauma to isolate the temporal integrity of the BBB during CSD and periorbital allodynia. Furthermore, we found increased uptake of ^3^H-sumatriptan in both the cortex and, contrary to ^14^C-sucrose studies, the brainstem in response to KCl-induced CSD (findings summarized in Fig. 6). This observation suggests the possibility for changes in active transport in the brainstem of triptans in response to CSD events. Therefore, the efficacy of these anti-migraine drugs may be partially dependent on increases in active transport mechanisms to reach central sites of action during CSD events. Together, our results support the dynamic nature of the BBB, during CSD events the paracellular integrity of the BBB is compromised and in turn may contribute to headache pathology and anti-migraine drug access to the CNS.

### Behavioral analysis of CSD-induced nociceptive behaviors

Studies examining the influence of CSD on nociceptive behaviors and BBB changes have used male rats to assess these parameters (Gursoy-Ozdemir et al., 2004; Fioravanti et al., 2011; Olah et al., 2013; Zhao and Levy, 2016). It is known, however, that migraine and many of the underlying disorders leading to secondary headache production afflict women more than men (Hu et al., 1999; Haast et al., 2012). A higher percentage of women migraineurs are also reported to seek medical care, and use triptans, more often than men (Brusa et al., 2015). Additionally, it has been suggested that women are more susceptible to secondary headache production in both traumatic brain injury and ischemic stroke compared to males (Tentschert et al., 2005; Lucas et al., 2012).

Head pain is the most prominent feature of both primary and secondary headaches, however, use of anesthesia in previous studies has precluded assessment of periorbital allodynia when studying changes in BBB integrity during CSD. Using surgically implanted guide cannulae, we circumvented the need for anesthesia when applying KCl to the cortex (Fioravanti et al., 2011). Following surgery, we observed significant postsurgical facial allodynia in our female rats, which was not paralleled in studies done in male rats (Fioravanti et al., 2011). Clinically, females have been observed to exhibit longer-lasting postsurgical pain with a higher intensity than males (Morin et al., 2000; Storesund et al., 2016). Following cortical KCl injection, the production of CSD events was confirmed with electrophysiological recordings in awake, freely moving rats. Furthermore, significant periorbital allodynia developed within 0.5 h post-KCl injection and peaked after 1.5 h. Although withdrawal thresholds improved close to baseline levels within 6 h, thresholds remained statistically lower up to 72 h post-KCl injection. Notably, the time of BBB opening corresponded to the peak duration of periorbital allodynia as assessed behaviorally. Non-evoked measures of nociceptive behavior such as rearing have also been used to assess responses in preclinical models of headache (Sandweiss et al., 2017; Zhang et al., 2017). Decreases in these non-evoked exploratory behaviors have been suggested to indicate a decrease in quality of life in these animals. In these studies rats injected with cortical KCl had decreased rearing activity, indicating general discomfort and stress in these animals. These data provide further evidence for CSD as a contributing event in the development of headache pain in primary and secondary headache disorders. Importantly, no significant periorbital allodynia was seen in rats given a cortical aCSF injection, indicating that these changes were due to KCl application and not the injection itself. Therefore, our current studies in female rats recapitulated clinical observations in headache populations to validate our model.

### BBB changes in response to CSD

One third of migraineurs experience focal neurologic symptoms known as aura (Silberstein, 2001). CSD originating in the occipital lobe is thought to contribute to the initiation of the aura phase of migraine with aura (Leo, 1944; Leao, 1947; Somjen, 2001). Aura is highly variable between patients and may have sensory, motor, verbal, auditory, or olfactory components, suggesting generalized cortical dysfunction. Onset of aura can occur during the premonitory phase and persist throughout the headache phase (Goadsby et al., 2017). Interestingly, numerous clinical studies have shown that sumatriptan, the most common drug used for the treatment of migraine, is most effective when given in the premonitory/aura phase or soon after the start of the headache phase (Bigal and Lipton, 2008; Aurora et al., 2009; Bigal et al., 2009; Gilmore and Michael, 2011; Thorlund et al., 2014; Martelletti, 2015). These observations suggest the possibility for changes in drug delivery dynamics to occur over the course of migraine progression. Studies assessing the CSD contribution to altered BBB integrity have shown increased paracellular leak following CSD induction (Gursoy-Ozdemir et al., 2004; Olah et al., 2013), although previous changes in BBB integrity in the context of CSD were performed in highly invasive models in anesthetized rats. In our model, we observed increased paracellular leak with a short onset latency (0.5 h) and fast resolution (within 6 h). The time points at which we observed BBB leakiness differ from a previous study (Gursoy-Ozdemir et al., 2004) where increased permeability to Evan’s Blue was seen up to 24 h following CSD induced with cortical pinprick. Several components in our model may account for these differences. One possibility is that we reduced the contribution of surgical trauma to BBB measurements in our model via dural cannula placement. In the studies done by Gursoy-Ozdemir et al. (2004), an exaggerated BBB response to CSD may have occurred due to ongoing surgical trauma resulting from use of cranial windows and dural retraction; these stresses may diminish barrier function alone (Spong et al., 2014). Additionally, our use of female rather than male rats may account for our observed differences; for instance, it has been shown that female sex hormones are protective against BBB disruption and repress MMP production (Cipolla et al., 2009; Khaksari et al., 2011; Na et al., 2015; Maggioli et al., 2016). On the other hand, we observed significant postsurgical facial allodynia and a more exaggerated nociceptive response to KCl in our female rats compared to those reported in male rats (Fioravanti et al., 2011). Therefore, it is possible that, due to hormonal effects, our female rats experienced both a more intense nociceptive response, leading to both a rapid onset and resolution of BBB disruption following KCl-induced CSD. These data suggest the possibility for differing mechanisms of increased BBB permeability in response to CSD in male versus female subject. Despite these differences in conditions, our data support increased paracellular permeability of the BBB following KCl-induced CSD events, which may influence drug delivery to the CNS.

The brainstem contains several processing centers important for the transmission and modulation of pain, including headache (Ossipov et al., 2010). For example, the second order neurons responsible for receiving nociceptive information from peripheral trigeminal nociceptors are in the TNC in the brainstem (Hoskin et al., 2001; Liu et al., 2009). Therefore, changes in permeability at the brainstem, an important target area for headache therapeutics, may be particularly relevant for drug delivery during headache. Although studies done in an inflammation induced model of headache showed changes in BBB permeability in the brainstem, namely the TNC (Fried et al., 2017), our studies revealed that cortical KCl injection did not increase paracellular permeability in the brainstem as measured by ^14^C-sucrose. This suggests the possibility of model-specific alterations in BBB permeability. One possible explanation for this is that KCl-induced CSD does not spread to the brainstem. In fact, it has been demonstrated that the brainstem is resistant to spreading depression originating from the cortex (Andrew et al., 2017).

Some clinical studies have also examined BBB changes in response to episodic headache, particularly migraine. These studies, however, have produced conflicting results. Increased MMP-9 activity was observed in one study during migraine headache attacks, indicating a breakdown of the BBB (Leira et al., 2007). Conversely, a PET imaging study done during glyceryl trinitrate (GTN)-induced migraine showed no increase in BBB permeability to ^11^C-dihydroergotamine (Schankin et al., 2016). This study was performed in an artificial induction model, however, which may not produce all of the complex features of migraine. Additionally, a recent MRI study demonstrated no change in BBB permeability in spontaneous attacks of migraine with aura (Hougaard et al., 2017). Importantly, this study represented the first highly sensitive imaging assessment of spontaneously occurring migraine with aura. Though the data from this clinical study suggest an opposing conclusion from those presented here, some differences in experimental parameters may explain these discrepancies. First, the timing of observation was significantly different from our studies. The average time from onset of aura to imaging in the MRI study was 7.6 h (Hougaard et al., 2017). In our study, however, we observed an immediate increase in BBB permeability which resolved very quickly (within 6 h). It is therefore possible that this clinical MRI study missed the time period corresponding to increased barrier permeability. Additionally, this MRI study consisted of 19 patients, which were genetically diverse and mixed-sex (Hougaard et al., 2017). Since migraine disorders are complex and, likely, arise from a multitude of different origins, it is possible that increased BBB permeability is not a feature which occurs in all migraine with aura variants. Still, based on other clinical studies as well as our own data, it is likely that increased BBB permeability occurs in at least some variants of migraine as well as in other primary or secondary headache disorders.

### Changes in CNS uptake of migraine therapeutics

Migraine-specific medications are used to combat migraine, with emphasis on moderate to severe migraine (Bigal et al., 2009; Martelletti, 2015; Starling and Dodick, 2015). Triptans are first-line therapies for episodic migraine associated with high therapeutic gains and favorable side-effect profiles (Humphrey et al., 1990; Ferrari, 1998; Gilmore and Michael, 2011; Smitherman et al., 2013; Thorlund et al., 2014). These serotonin (5HT) derivatives constitute 80% of migraine prescriptions (Smitherman et al., 2013); sumatriptan represents ∼50% of the market (Smitherman et al., 2013). Only ∼30% of patients have reduced head pain intensity and duration with triptan compounds (Malone et al., 2015; Smitherman et al., 2013; Burgos-Vega et al., 2015). Importantly, migraineurs report that triptans need to be on-board during prodrome for effect and that triptans are less effective when taken at the headache phase (Bigal and Lipton, 2008; Bigal et al., 2009; Gilmore and Michael, 2011; Thorlund et al., 2014; Martelletti 2015) suggesting an unknown pharmacokinetic/dynamic mechanism of action that may relate to BBB/NVU integrity. Using ^3^H-sumatriptan, a drug not typically thought to cross the BBB, we again observed increased BBB leakiness in the cortex following cortical KCl injection. Surprisingly, and in contrast with our data with ^14^C sucrose, we saw a robust increase in brainstem uptake of ^3^H-sumatriptan 1.5 h post-KCl injection. This disparity between ^14^C-sucrose and ^3^H-sumatriptan in the brainstem may be explained by changes in active transport. While sucrose has no known endogenous transporter, sumatriptan has at least one putative transporter, the influx transporter organic anion-transporting polypeptide (OATP) 1A2 (rat Oatp1a4), although its role in transporting sumatriptan to the CNS has yet to be elucidated (Cheng et al., 2012). Furthermore, increases in the function and expression of Oatp1a4 have been observed at the BBB in response to inflammatory pain as well as hypoxia/reoxygenation stress (Ronaldson et al., 2011; Thompson et al., 2014). It has yet to be determined, however, whether migraine or other headache disorders produce similar changes in brainstem Oatp1a4/OATP1A2. Further investigation is warranted, to determine the exact cause of increased sumatriptan uptake in the brainstem following cortical KCl injection. The observed increase in brainstem uptake of sumatriptan may represent an opportunity for triptans such as sumatriptan, which do not normally cross to the CNS in appreciable levels to exert a therapeutic effect in these relevant brainstem areas. Therefore, with the proper timing of dosing, these drugs may be able to achieve a greater therapeutic efficacy in the indicated migraine population with expanded use to secondary headache disorders.

Topiramate is an anti-epileptic which is also used as a migraine prophylactic. Topiramate functionally modulates voltage gated sodium and calcium channels, GABA receptor potentiation, and blockade of glutamatergic transmission (Akerman and Goadsby, 2005). Important to this study, a single application of topiramate has been shown to block CSD initiation (Akerman and Goadsby, 2005; Unekawa et al., 2012; Green et al., 2014). We observed that topiramate pretreatment was not able to block KCl-induced BBB leakiness. Additionally, periorbital allodynia was unaffected with topiramate pretreatment. Since topiramate therapy for migraine is often prophylactic to reduce migraine attack frequency and magnitude (Silberstein et al., 2004; Martelletti, 2015; He et al., 2017; Silberstein, 2017) and not abortive, it is possible that repeated administration of topiramate is required to have effects on periorbital allodynia in this model. Thus, present findings suggest that regulation of BBB integrity and nociception in response to a single KCl injection arise concurrently from separate, and likely complex, mechanisms.

### Concluding remarks

Disorders resulting in primary and secondary headache production arise from distinct etiologies. Although their pathologies are varied, the development of CSD remains a common event among them (Olesen et al., 1990; Hadjikhani et al., 2001; Bolay et al., 2002; Dreier et al., 2006; Hartings et al., 2009; Zhang et al., 2010; Lauritzen et al., 2011; Woitzik et al., 2013; Chen and Ayata, 2016). In already compromised tissue (i.e., in ischemic stroke), CSD has been demonstrated to facilitate neuronal death (Dreier and Reiffurth, 2015; Androulakis et al., 2016). Conversely, CSD occurring in otherwise “normal” tissue, such as in migraine with aura, is seen as relatively benign; although it may contribute to an increased risk for stroke occurrence (Santos et al., 2012). Changes in BBB permeability have been shown in numerous disease states to further contribute to CNS damage (Friedman and Kaufer, 2015). On the other hand, transient disruptions in BBB permeability resulting from conditions such as migraine with aura/CSD may provide an opportunity to facilitate drug delivery to the CNS, potentially improving disease outcomes. In conditions such as TBI and ischemic stroke, the integrity of the BBB is altered following injury (Prakash and Carmichael, 2015), although this loss of integrity is most often associated with trauma within the CNS rather than with CSD or headache.

This study represents a novel, parallel observation of both nociceptive behavior and BBB changes in a minimally invasive model of CSD induction in awake, freely moving rats. Overall, the data presented here supports the proposition of increased BBB permeability and compromised integrity of the microvasculature in response to CSD. Furthermore, our studies suggest that these changes influence CNS uptake of the abortive ant-migraine agent, sumatriptan. The ability of drugs to penetrate the BBB remains the most significant obstacle in the development of drugs for disease with a CNS component. Previous reports have shown little to no CNS penetration of sumatriptan (Ahn and Basbaum 2005). The data presented here, however, suggests an enhanced uptake of sumatriptan to the CNS following CSD and headache production that is temporally dependent, potentially leading to alterations in therapeutic efficacy. Therefore, this study highlights the importance of uncovering pathologic regulation of BBB integrity, to facilitate development effective therapeutic regimens for CNS mediated pathologies. Based on these findings, further investigation is warranted to determine the precise mechanisms driving the BBB changes observed response to KCl-induced CSD.

## Synthesis

Reviewing Editor: Dritan Agalliu, Columbia University Medical Center Decisions are customarily a result of the Reviewing Editor and the peer reviewers coming together and discussing their recommendations until a consensus is reached. When revisions are invited, a fact-based synthesis statement explaining their decision and outlining what is needed to prepare a revision will be listed below. The following reviewer(s) agreed to reveal their identity: Martha O'Donnell.

The authors present a timely, well-designed and well-executed study of BBB changes occurring in response to KCl-induced CSD events. The manuscript is quite well written. The authors propose that cortical spreading depression (CSD) compromises the blood brain barrier which in turn allows sumatriptan to access targets in the brain. They infuse the cortex of female rats with KCl, which results in increased periorbital allodynia, and on a similar time scale increases blood-brain barrier (BBB) permeability as measured by 14C-sucrose. They also find that KCl infusion increases permeability to 3H-sumatriptan, a well established migraine abortive treatment.

Reviewer 1:

The conclusion that CSDs increase BBB permeability thus increasing effectiveness of migraine medications is an interesting idea. However, it is premature with the data presented.

Major concerns for the study are as follows:

1) The authors don't show that their protocol of cortical KCl infusion induces CSD. There's a sentence at the beginning of the results section, but it would be important to actually show the data - especially if they conclude that CSD is responsible for increased BBB permeability.

2) The methods indicate that estrous cycle was monitored, but there is no comment in the results or discussion about how it is related to the rest of the data. This is important to address in the study.

3) There is increased 3H-sumatriptan in the brain following KCl infusion. However, the authors do not demonstrate that sumatriptan is effective at inhibiting periorbital allodynia within their model.

4) There needs to be a discussion about the work from Messoud Ashina's group showing a lack of change in BBB in migraine patients with and without aura.

5) What method was used to test von Frey hair stimulation?

Reviewer 2:

1. Methods: In the paragraph on Western blot, it would be helpful to include information about what was done with the tissue samples after centrifugation prior to loading on the gels, i.e., was the pellet re-suspended? In what?

2. Results: Paragraph on BBB permeability increase following KCl injection and Figure 2/Figure 2 legend. It is not obvious when first reading the legend that Figure 2 A is for cortex and Figure 2 B is for brainstem. Please clarify this in the figure legend. I recommend making this clarification throughout the manuscript where pertinent.

3. For readers not familiar with KCl induction of cortical spreading depression it would be helpful to clarify the amount of KCl injection and how this leads to CSD. 0.5 µl of 1 M KCl would be an injection of only 0.5 µmol, correct? Given that extracellular [K] is on the order of 3 mM that does not seem sufficient to cause CSD. Please explain this in greater detail and demonstrate that this amount causes CSD.

4. Figure 3: It appears that Figure 3A and 3B have been switched. The legend indicates that Occludin data are shown in A and Claudin-5 data in B.

4. Figure 4: With regard to sumatriptan permeability, it would be good to provide the molecular size and how that compares to sucrose regarding the diffusion across the BBB.

5. Discussion and Figure 6 legend: The legend to Figure 6 states that increased entry of sumatriptan is likely via paracellular leak. However, the discussion text also mentions active transport. Please clarify that active transport is only proposed for brainstem. This is confusing as presented and to be discussed in greater detail.

## Author Response

Ref: revision of eNeuro eN-NWR-0116-18

Title: Loss of Blood-Brain Barrier Integrity in a KCl-Induced Model of Episodic Headache Enhances CNS Drug Delivery

The authors wish to thank the reviewers for their remarks and support for the research/manuscript. Below we have addressed individual concerns/comments in bold. Within the text of the manuscript, we have highlighted revision in red font.

Reviewer 1: critique and response to review Overall issue: The conclusion that CSDs increase BBB permeability thus increasing effectiveness of migraine medications is an interesting idea. However, it is premature with the data presented.

Thank you for assessing the overall issues with this manuscript and detailing the specific concerns that need to be addressed. We feel that, with our included edits based on your feedback, the conclusions presented in this manuscript are now well supported based on our data and the accompanying text.

Major concerns for the study are as follows: 1) The authors don't show that their protocol of cortical KCl infusion induces CSD. There's a sentence at the beginning of the results section, but it would be important to actually show the data - especially if they conclude that CSD is responsible for increased BBB permeability.

Thank you for bringing this to our attention. We have added a table (Table 1) which details variables relevant to CSD events from our assessment including EEG percent decrease, frontal and parietal amplitude decrease (mv), and duration (s). This data is represented as mean ± SEM and corresponds to the CSD event inclusion parameters as described in the methods section. We have also included the range of time in the text for which KCl-induced CSD events occurred post cortical injection.

2) The methods indicate that estrous cycle was monitored, but there is no comment in the results or discussion about how it is related to the rest of the data. This is important to address in the study.

Thank you for catching this omission. A line has been added in the methods section to state: “Cortical injections were given during the diestrus phase of the estrous cycle. CSD events, pain behaviors, and BBB permeability were also

2

assessed during the diestrus phase; except in the case of multiple day assessments where rats continued to progress through the estrus cycle”. 3) There is increased 3H-sumatriptan in the brain following KCl infusion. However, the authors do not demonstrate that sumatriptan is effective at inhibiting periorbital allodynia within their model.

Due to the nature of the in situ brain perfusion model, behavioral assessment during compound perfusion is not possible. The increased 3H-sumatriptan in these studies represents a proof of concept that increased BBB uptake of a permeability marker (14C-sucrose) can correspond to changes in drug uptake as well. An initial behavioral assessment where sumatriptan was dosed 10 min prior to cortical KCl injection did indicate significant anti-allodynic efficacy in the KCl model similar to other published studies (Kayser et al. 2002, Yao et al. 2008, Tomic et al. 2015, Sufka et al. 2016, Sandweiss et al. 2017). 4) There needs to be a discussion about the work from Messoud Ashina's group showing a lack of change in BBB in migraine patients with and without aura.

The authors recognize the importance of the Hougaard et al. paper from the Ashina group to the field. We have since expanded our discussion section further to consider their observations in the context of the present findings. This paragraph is located under the “Blood brain barrier changes in response to CSD” sub-heading on page 21. 5) What method was used to test von Frey hair stimulation?

An additional sentence was added to the methods section to clarify the method of von Frey assessment: “The withdrawal threshold was determined using a modified version of the Dixon up-down method, where the response pattern was used to obtain the threshold as reported by Chaplan et al.(Chaplan et al. 1994).”

Reviewer 2: critique and response to review 1. Methods: In the paragraph on Western blot, it would be helpful to include information about what was done with the tissue samples after centrifugation prior to loading on the gels, i.e., Was the pellet re-suspended? In what?

The text for this portion of the methods was revised to clarify that centrifugation was utilized to remove solid particles from the tissue lysate. The tissue supernatant in the collection buffer was used for the gel loading. This portion now reads: “Tissue samples were prepared with inhibitors as described above. Samples were incubated for 10 min at 70°C and centrifuged for 5 min at 13,000 × g

3

at 21°C to separate any solid particles prior to gel electrophoresis. 10 µg of total proteins from the tissue supernatant were loaded on TGX precast gels...” 2. Figure 2/Figure 2 legend. It is not obvious when first reading the legend that Figure 2 A is for cortex and Figure 2 B is for brainstem. Please clarify this in the figure legend. I recommend making this clarification throughout the manuscript where pertinent.

Thank you for bringing this to our attention, all figures have been edited so that graphs corresponding to cortex or brainstem can be easily distinguished.

3. For readers not familiar with KCl induction of cortical spreading depression it would be helpful to clarify the amount of KCl injection and how this leads to CSD. 0.5 µl of 1 M KCl would be an injection of only 0.5 µmol, correct? Given that extracellular [K] is on the order of 3 mM that does not seem sufficient to cause CSD. Please explain this in greater detail and demonstrate that this amount causes CSD. This is a critical aspect of the study.

The text has been edited to state that the high concentration of KCl induces CSD by disrupting the extracellular ion equilibrium. It should be noted that in switching molarity to moles, although 0.5µl of 1M KCl is equivalent to 0.5µmol an equal volume of 3mM potassium (physiologic [K+]) would equate to 0.0015µmol (3 mmol/L in 0.5µl), which is significantly less than what we delivered cortically. During CSD events, extracellular [K] has been reported to be as high as 55mM (reviewed by Ayata and Lauritzen, 2015; Olesen et al., 2009; Pietrobon and Moskowitz, 2014, Hansen et al., 1981).

4. Figure 3: It appears that Figure 3A and 3B have been switched. The legend indicates that Occludin data are shown in A and Claudin-5 data in B.

Thank you for pointing out this error, the revised figure has been corrected.

4. Figure 4: With regard to sumatriptan permeability, it would be good to provide the molecular size and how that compares to sucrose regarding the diffusion across the BBB.

The text has been updated to include the molecular weights of both compounds (sucrose= 342.30 g/mol, sumatriptan = 295.402 g/mol). Both compounds are of similar size, with sucrose being slightly larger.

4

5. Discussion and Figure 6 legend: The legend to Figure 6 states that increased entry of sumatriptan is likely via paracellular leak. However, the discussion text also mentions active transport. Please clarify that active transport is only proposed for brainstem. This is confusing as presented and to be discussed in greater detail.

The text has been edited to further clarify that active transport is currently, only proposed in the brainstem. Figure 6 has also been edited to clarify that increased active transport is proposed in the brainstem

References:

Chaplan, S. R., F. W. Bach, J. W. Pogrel, J. M. Chung and T. L. Yaksh (1994). “Quantitative assessment of tactile allodynia in the rat paw.” J Neurosci Methods 53(1): 55-63.

Kayser, V., B. Aubel, M. Hamon and S. Bourgoin (2002). “The antimigraine 5-HT 1B/1D receptor agonists, sumatriptan, zolmitriptan and dihydroergotamine, attenuate pain-related behaviour in a rat model of trigeminal neuropathic pain.” Br J Pharmacol 137(8): 1287-1297.

Sandweiss, A. J., K. E. Cottier, M. I. McIntosh, G. Dussor, T. P. Davis, T. W. Vanderah and T. M. Largent-Milnes (2017). “17-beta-Estradiol induces spreading depression and pain behavior in alert female rats.” Oncotarget 8(69): 114109-114122.

Sufka, K. J., S. M. Staszko, A. P. Johnson, M. E. Davis, R. E. Davis and T. A. Smitherman (2016). “Clinically relevant behavioral endpoints in a recurrent nitroglycerin migraine model in rats.” J Headache Pain 17: 40.

Tomic, M. A., U. B. Pecikoza, A. M. Micov, B. V. Popovic and R. M. Stepanovic-Petrovic (2015). “The effects of levetiracetam, sumatriptan, and caffeine in a rat model of trigeminal pain: interactions in 2-component combinations.” Anesth Analg 120(6): 1385-1393.

Yao, D. and B. J. Sessle (2008). “Nitroglycerin facilitates calcitonin gene-related peptide-induced behavior.” Neuroreport 19(13): 1307-1311.
